# Assessment of Knowledge, Attitude and Barriers towards Pharmacovigilance among Physicians and Pharmacists of Abbottabad, Pakistan

**DOI:** 10.3390/pharmacy6020029

**Published:** 2018-03-31

**Authors:** Akash Syed, Saira Azhar, Muhammad Mohsin Raza, Humaira Saeed, Shazia Qasim Jamshed

**Affiliations:** 1 Department of Pharmacy, COMSATS Institute of Information Technology, Abbottabad 22060, Pakistan; drakashsyed@gmail.com (A.S.); drmmohsinraza@gmail.com (M.M.R.); humairasaeed17@yahoo.com (H.S.); 2 Department of Pharmacy Practice, College of Pharmacy, Princess Nourah bint Abdulrahman University, Riyadh 84428, Saudi Arabia; SAAzhar@pnu.edu.sa or drsairaazhar@gmail.com; 3 Department of Pharmacy Practice, Kulliyyah of Pharmacy, International Islamic University Malaysia, Kuantan 25200, Malaysia

**Keywords:** pharmacovigilance, ADR, pharmacist, physician

## Abstract

Objectives: Pharmacovigilance in Pakistan needs robust preference in terms of implementation and consistent movement of structured approaches. The objective of this study is to explore the knowledge, attitude and barriers towards adverse drug reaction (ADR) reporting among physicians and pharmacists and to explore the encouraging factors of ADR reporting. Methods: The current research was a cross-sectional study design in which a pre-validated questionnaire was administered to physicians and pharmacists in Abbottabad, Pakistan. The study was conducted for two months from January 2016 to February 2016. Results: A total of 194 physicians and pharmacists responded with a response rate of 35.3%. All the respondents either strongly agreed or agreed that ADRs reporting is a part of their duty. Half of the respondents agreed that monitoring of drug safety is important. Around three quarters of respondents (74.2%) stated that they did not report ADRs due to unavailability of reporting forms while 70% cited lack of a proper pharmacovigilance center as one of the key barriers. Half of the respondents (52.2%) did not report due to their insufficient knowledge. A large majority (81.8%) said that they would report ADRs if there is pharmacovigilance center. On the point of incentives, opinion seems to be divided. Slightly less than half (47.8%) cited their wish to have few incentives while the remaining 52.2% either preferred to be neutral or disagreed. Conclusion: Based on the study findings, barriers were mostly related to general unfamiliarity with ADRs reporting guidelines and the non-existence of a pharmacovigilance center. It is highlighted that the regulatory body should carve a niche for a properly functional pharmacovigilance center and initiate educational programs for strengthening knowledge and attitudes towards ADR reporting.

## 1. Introduction

Whenever one discusses pharmacovigilance and principles of reporting it is impossible to ignore the thalidomide catastrophe. In fact, the thalidomide disaster after the Second World War was the main reason for the World Health Organization (WHO) to introduce the Program for International Drug Monitoring (PIDM), preferentially for early detection of ADRs. This activity is termed as pharmacovigilance and is known as, “science and activities related to the detection, assessment, understanding and prevention of adverse effects or any other possible drug-related problems” [1]. The WHO aims to strengthen this concept globally and thereby promotes pharmacovigilance at every country level and thus, 134 countries are working conjointly with the Monitoring Center, Uppsala, Sweden. The program not only enhances patient safety for the use of medicines but also gives information about the safe use of drugs and the prevention and treatment of any Adverse Drug Reactions (ADRs) [1]. ADRs occur during drug therapy and most of them are preventable with proper management [2,3]. ADR monitoring is a vital task of the quality assurance department in developed nations [4] but unfortunately Pakistan has a limited accountability system for medicines. The actual number of deaths related to ADRs is not known due to an underdeveloped process of pharmacovigilance. A couple of hospitals practicing ‘state of the art’ pharmacy services—namely Aga Khan University Hospital and Dow University Hospital—are providing localized pharmacovigilance services. However, rationalized efforts are initiated for a pharmacovigilance center [5]. In this context, one cannot put aside the services of the Pakistan Pharmacist Federation, which initiated a campaign on the prerogative of the Health Department, Government of Punjab, Pakistan to establish pharmacovigilance centers, ADRs reporting, and Drug Information and Poison Control Centers at the provincial and hospital level [6].

Pharmacovigilance is gaining momentum as a new aspect of pharmaceutical sciences and is critically important in public health and clinical practice. Underreporting of ADRs by health care professionals affects the public health domain [7]. Many factors are responsible for underreporting of ADRs. Inman has designated them as “seven deadly sins” comprised of: *Financial incentives*: Rewards for reporting; *legal aspects*: Fear of enquiry and prescribing data compilation; *complacency*: ADRs are well documented by the time a drug is promoted; *diffidence*: Only reporting ADRs that are surely caused by particular drug; *indifference*: Only a single case observed by me cannot improve medical knowledge; *ignorance*: Confidence that it is necessary to report serious and unexpected ADRs; and *lethargy*: Delay in reporting or lack of time to find a report card and other excuses [8].

As per WHO standards, countries with the best reporting rates generate over 200 reports per 1,000,000 inhabitants per year. In Pakistan, with a population of over 200 million, at least 40,000 reports per year would be expected but this was not observed. In a recent declaration, the Drug Regulatory Authority of Pakistan (DRAP) announced the establishment of a National Pharmacovigilance Center to streamline unremitted monitoring of marketed medicines and therefore, better results can be foreseen in terms of reporting.

The participation of health care professionals in spontaneous reporting is expected to be improved by strengthening equally the intrinsic factors (knowledge, attitude and practices) and extrinsic factors (relationship between health professionals and their patients, the health system and the watchdogs) [9]. The knowledge and attitude of health care professionals are core factors that need extensive exploration in this regard. Therefore, the aim of the current research is to determine the knowledge, attitude and barriers towards ADRs reporting in the physicians and pharmacists of Abbottabad, Pakistan as well as to distinguish the factors that could strengthen reporting of ADRs.

## 2. Materials and Methods

### 2.1. Study Participants and Sampling

The sampling frame included all the physicians and pharmacists who were practicing in the Abbottabad KPK (Ayub Medical Complex, District Headquarters DHQ Abbottabad). Total numbers of physician and pharmacist data were obtained from the administration cell of Ayub Medical Complex and DHQ.

### 2.2. Study Design, Setting and Recruitment of Participants

The survey was conducted from 1 January 2016 to 29 February 2016. A total of 194 physician and pharmacists responded to the study. A self-administered questionnaire was used. Self-administered instruments are most frequently used methods for collection of data in research, where respondents complete the questionnaires by themselves [10]. There are two types of self-administered questionnaires: supervised and unsupervised. The former involves people answering in the presence of the researcher and the latter permits the respondents to complete the questionnaires by themselves [11]. In the present study, the questionnaire was distributed among the eligible practicing physicians and pharmacists with an explanatory note stating the purpose of research. Two weeks after the distribution the principal investigator collected the questionnaires back.

The responders were assured of the confidentiality of their details and all forms were coded to ensure anonymity. No compensation was given to the respondents for their participation in the current research.

### 2.3. Development of the Survey Questionnaire

The initial research instrument was developed using information from the previously published literature in the area of ADR and ADR reporting, principally highlighting the attitudes, perceptions and barriers towards ADR reporting among physicians, pharmacists and other healthcare professionals [12,13,14,15,16,17]. The questionnaire consisted of 43 items which cover the following areas of interest:

The first part consisted of 13 items which covered respondents’ demographics, sources of information or reference materials available on pharmacies/clinics and continuous educational activity for healthcare professionals. These items were posed as either questions or multiple-choice options.

The second part consisted of 12 items exploring the respondents’ attitudes towards reporting and the factors that either positively or negatively affect the respondents’ attitudes. These items were worded as a series of statements and the respondents were asked to show their agreement or disagreement on a five-point. Likert scale (1 = “strongly agree,” 2 = “agree,” 3 = “neutral,” 4 = “disagree” and 5 = “strongly disagree”).

The third part consisted of 12 items which further explored the barriers for not reporting ADRs. These statements also adopted a five-point Likert scale format.

The fourth part of the questionnaire consisted of six items aimed at identifying the factors that possibly encourage and motivate the physicians and pharmacists to report ADRs. These items were also framed in a five-point Likert scale. All the items evaluated by the Likert scale were awarded both positively and negatively within each part to avoid acquiescence, affirmation or agreement bias [18,19,20,21].

### 2.4. Content and Face Validity

Prior to the survey, the questionnaire was piloted with a convenience sample of 20 respondents, who were excluded from the main study. This resulted in the adaptation of the phrasing of several of the questions and the reliability of the questionnaire was assessed using Cronbach’s coefficient alpha [22]. The overall internal consistency of the instrument was 0.798.

### 2.5. Data Analysis

Data was analyzed using SPSS v23 (IBM, New York, NY, USA) and Microsoft Excel. Frequencies and percentages were expressed through descriptive statistics. Associations between the groups were examined by applying inferential statistics [23,24]. Data which emerged from domains using the Likert scale as a measurement emerged as non-parametric data [25,26]. However, the response categories in the Likert scale have a rank order but the intervals between values cannot be presumed equal [27]. Values of *p* < 0.05 considered statistically significant at confidence interval 95%.

### 2.6. Ethical Consideration

The study received exemption status from Research Committee of COMSATS Abbottabad, Pakistan. Participants were appraised about the nature of the study and informed consents were signed by the participants.

## 3. Results

### 3.1. Response Rate

During the period of two months from 1 January 2016 to 29 February 2016, a total of 194 physician and pharmacists responded to the study. The response rate based on the number of doctors (*n* = 467) and pharmacists (*n* = 82) in Abbottabad was found to be 35.3%. The response rate for physician was 29.1% while for pharmacist it was 56%. The total number of usable questionnaires were 182.

### 3.2. Demographics

The majority of the respondents were in the age group of 30 to 40 years old (*n* = 87, 47.8%) accounted as the biggest group. Majority of the respondents were physicians (*n* = 131, 72%) and were employees (*n* = 117, 64.3%). Most commonly used reference was British National Formulary (BNF) (*n* = 105, 57.7%) followed by Pakistan Drug Manual (*n* = 67, 36.8%). Only a very few (*n* = 10, 5.5%) used other sources of drug information. Detailed demographics are shown in Table 1.

### 3.3. Attitude towards ADRs Reporting

Respondents when asked whether reporting ADRs was a part of physicians’ or pharmacists’ duties and in response 73% (*n* = 96) of physicians agreed that ADRs reporting is part of a physician’s duty and 76.4% (*n* = 39) of pharmacists also agreed that ADRs reporting is part of a pharmacist’s duty. Statistically, there was a significant association between this statement and gender (*p* = 0.047) and age (*p* = 0.044) and greater association was noted in the male respondents of 30–40 years of age.

Half of the respondents 52.2% (*n* = 95) strongly agreed that the monitoring of drug safety is important. Respondents who were employees (*p* = 0.001) and in the age range of 30–40 years (*p* = 0.001) showed greater concern towards drug safety. Slightly less than half of the respondents (*n* = 87; 47.8%) expressed no need to report ADRs related to OTC products. Employees (*p* = 0.001) in between 30 and 40 years (*p* = 0.001) observed to have more significant opinions than other respondents. A large majority (*n* = 134; 73.6%) expressed their agreement towards the improvement of pharmacovigilance by delegating the responsibility to academia or pharmaceutical industry. This is more significantly observed in employees (*p* = 0.005) rather than managers or sole proprietors. Furthermore, nearly half of the respondents (*n* = 87; 47.8%) expressed the need to discuss ADR report with the physician or academician trained in this field. This is also significantly observed in employees (*p* = 0.001) of 30–40 years of age (*p* = 0.001). For detailed results please refer to Table 2.

### 3.4. Barriers to ADRs Reporting

In this section, a total of 12 statements explored the barriers generally perceived by respondents in terms of ADRs reporting (Table 3).

One of the main factors which strongly discouraged respondents (*n* = 182; 74.2%) from ADR reporting is the unavailability of reporting forms. On the contrary, in the presence of their availability, a majority (51.6%) expressed their inability to complete them as they were not aware about how to report. This is significantly observed in male (*p* = 0.001) employees (*p* = 0.006) within 30–40 years of age (*p* = 0.001). Refer to Table 3 for detailed results.

### 3.5. Factors Encouraging Physicians/Pharmacists towards ADR Reporting

In this section, a total of six questions were asked to identify the factors, which might encourage physicians/pharmacists to report ADRs (Table 4).

More than three-fourth respondents (*n* = 149; 81.8%) stated that they would report ADRs if there is proper pharmacovigilance center in Pakistan. This showed statistical significance with respect to type of practice (*p* = 0.037) and age (*p* = 0.008) and greater association was noted among the managers of 30–40 years old. However, slightly less than of the respondents (*n* = 87; 47.8%) mentioned their interests to incentivize reporters and this was observed more significantly among the employees of 30–40 years of age (*p* = 0.001). whereas a large majority (81.3%) stated that they would be willing to report on the prompting of relevant authorities.

## 4. Discussion

In the current research a response rate of 35.3% was achieved. The study results demonstrate that a majority of the respondents have a positive attitude towards ADR reporting, even though a majority of them had never reported ADRs and this is in concordance with previously published research [28,29,30]. A majority of the respondents believed that the healthcare professional must be confident about the cause of ADR before reporting. These findings are consistent with the findings of previous studies [31,32,33,34]. More than half of the respondents believed that ADRs associated with OTC products should be reported. Similar findings were reported for pharmacists [17] and for physicians [13,35,36] in other countries.

Several studies have been carried out in developed and developing countries to ascertain the implicating factors of non-reporting [12,30,37,38,39,40,41]. The current research highlighted numerous factors of ADR underreporting in Pakistan. The majority of the healthcare professionals believed that the inaccessibility of ADR reporting forms and the absence of a national pharmacovigilance center are major issues for non-reporting. A majority in the current research believed that they have insufficient knowledge in the detection of ADRs. The findings are similar to studies conducted in India and Spain in which lack of knowledge about the reporting system was cited as a prominent factor followed by Japanese research, which also reported inadequate understanding of pharmacovigilance [9,39,42]. More than half of the respondents did not know how and where to report ADRs, similar to a study conducted in Southeastern Europe [43].

In this study, the majority of the respondents indicated that they would report if there was a proper pharmacovigilance center in Pakistan. More than half of the respondents indicated that their motivation to report would be increased if they received a letter from relevant authorities about reporting. This finding is similar to the findings reported in previous studies [14,40,43,44]. A recently published study from Yemen highlighted intense evidence of the under-reporting of ADRs and likewise, pharmacovigilance center in Yemen is suggested to strategize their efforts for the improvement of awareness of healthcare professionals on harm and benefits of medicines [45].

Last but not the least, another opinion of participants that is worth mentioning was about incentives. Slightly less than half agreed to have incentives for reporting ADRs while the remaining either preferred to be neutral or disagreed. This finding is in concordance with the recently published time series analysis from China, which advocated the effects of financial incentives on spontaneous ADR reporting [46].

Among the few limitations attributed to the current research, one is the low response rate when compared to similar studies conducted globally [12,14,28]. This low response rate may be related to lack of time and interest among health care professionals. Moreover, it is reported previously in the literature that non-response bias is a less concerning factor in physicians presumably because they are thought to be consistent in their opinions about attitudes, views, training and behavior [47].

Moreover, the current research is still valuable as even in developed ADR reporting cultures, few healthcare practitioners found to be engaged in ADR reporting and thus, this study shows a sizeable interest in pharmacovigilance and therefore, the views of participants involved in filling out the questionnaire are important.

## 5. Conclusions

The current research highlighted a lack of knowledge in terms of different aspects of pharmacovigilance but cited positive attitudes towards reporting. The barriers identified were a general unfamiliarity with ADRs reporting guidelines and therefore emphasis was placed on the need for a pharmacovigilance center. It is pertinent that regulatory bodies should launch a properly functioning pharmacovigilance center and arrange educational programs intended to intensify knowledge of ADR reporting. These goals can be achieved through cooperation with the local and/or international regulatory bodies involved in pharmacovigilance activities both at the academic and/or organizational levels.

## Figures and Tables

**Table 1:** Demographic descriptions of participants.

Description	*n*	%
Gender		
Male	136	74.7
Female	46	25.2
Age		
<30	85	46.7
30–40	87	47.8
41–50	10	5.4
Field of Practice		
Physician	131	71.9
Pharmacist	51	28.0
Duration of Practice in years		
≤5	96	52.7
6–10	76	41.7
>10	10	5.4
Type of Practice		
Sole proprietor	46	25.3
Manager	19	10.4
Employee	117	64.2
Reference Used		
BNF	105	57.6
Pakistan Drug Manual	67	36.8
Others	10	5.4

**Table 2:** Attitude towards Adverse Drug Reactions (ADRs) Reporting.

	Responses *					*p* Value **			
Statements	SA	A	N	D	SD	Age	Gender	Type of Practice	Direction of Association
	*n* (%)	*n* (%)	*n* (%)	*n* (%)	*n* (%)				
1	47 (25.8%)	135 (74.2%)	0	0	0	**0.044**	**0.047**	0.660	<30/Male
2	95 (52.2%)	87 (47.8%)	0	0	0	**0.001**	0.174	**0.001**	30–40/Employee
3	47 (25.8%)	135 (74.2%)	0	0	0	**0.001**	**0.047**	**0.003**	30–40/Male/Employee
4	40 (22%)	47 (25.8%)	47 (47.8%)	48 (26.4%)	0	**0.001**	0.076	**0.001**	30–40/Employee
5	135 (74.2%)	47 (25.8%)	0	0	0	0.054	**0.047**	0.660	Male
6	87 (47.8%)	95 (52.2%)	0	0	0	**0.001**	0.174	**0.001**	30–40/Employee
7	135 (74.1%)	47 (24.8%)	0	0	0	0.054	**0.047**	0.660	Male
8	47 (25.8%)	135 (74.2%)	0	0	0	**0.001**	**0.047**	**0.003**	30–40 Male/Employee
9	0	47 (25.8%)	87 (47.8%)	48 (47.8%)	0	0.084	0.142	**0.005**	Employee
10	135 (74.2%)	47 (25.8%)	0	0	0	**0.001**	**0.047**	**0.003**	30–40 Male/Employee
11	47 (25.8%)	87 (47.8%)	48 (26.4%)	0	0	0.084	0.142	**0.005**	Employee
12	0	87 (47.8%)	47 (25.8%)	48 (26.4%)	0	**0.001**	0.455	**0.001**	30–40/Employee

* Significance for comparison performed by the Mann-Whitney U test (to compare 2 groups) or the Kruskal-Wallis test (to compare >2 groups); * Reported responses were for all respondents; ** SD = Strongly disagree, D = Disagree, N = Neutral, A = Agree, SA = Strongly agree.
Reporting of ADRs is a part of pharmacist/physician duty. I believe that monitoring drug safety is important. It is necessary to confirm before reporting that an ADR is related to a particular drug. It is not necessary to report ADRs related to OTC products prescribed/dispensed in my clinic/pharmacy. It is important to report ADRs leading to hospitalization. It is important to report ADRs leading to a life-threatening situation. It is important to report ADRs leading to congenital abnormality. It is important to report ADRs leading to persistence disability or incapacity. It is important to report ADRs to answer the questions that may arise in my practice. Reporting of ADRs is important to show patients that their concerns are taken seriously. Moving the responsibility of pharmacovigilance scheme to pharmaceutical industry or academia will improve ADRs reporting. It is important to discuss ADRs with a physician and/or an academician trained in this aspect.

**Table 3:** Barriers to ADRs Reporting.

	Responses *					*p* Value **			
Statements	SA	A	N	D	SD	Age	Gender	Type of Practice	Direction of Association
	*n* (%)	*n* (%)	*n* (%)	*n* (%)	*n* (%)				
1	135 (74.2%)	47 (25.8%)	0	0	0	0.055	**0.047**	0.660	Male
2	47 (25.8%)	95 (52.2%)	40 (22%)	0	0	**0.001**	**0.001**	0.737	30–40/Male
3	0	7 (3.8%)	130 (71.2%)	45 (24.7%)	0	**0.001**	0.094	**0.005**	30–40/Employee
4	45 (24.7%)	84 (46.2%)	46 (25.2%)	4 (2.2%)	3 (1.6%)	0.075	0.184	**0.002**	Employee
5	0	47 (25.8%)	47 (25.8%)	88 (48.3%)	0	**0.003**	**0.001**	0.181	30–40/Male
6	0	42 (23.0%)	89 (48.9%)	48 (26.4%)	3 (1.6%)	**0.001**	0.164	**0.001**	30–40/Employee
7	87 (47.8%)	47 (25.8%)	0	48 (26.4%)	0	**0.001**	0.455	**0.001**	30–40/Employee
8	0	95 (52.2%)	47 (25.8%)	40 (22%)	0	**0.001**	**0.016**	**0.001**	Male/30–40/Employee
9	47 (25.8%)	47 (25.8%)	88 (48.3%)	0	0	**0.001**	**0.001**	**0.006**	Male/30–40Employee
10	47 (25.8%)	47 (25.8%)	48 (26.4%)	0	0	0.084	0.142	**0.005**	Employee
11	0	0	47 (25.8%)	95 (52.2%)	40 (22%)	**0.001**	**0.001**	0.063	30–40/Male
12	0	87 (47.8%)	68 (37.4%)	20 (11%)	7 (3.8%)	**0.005**	0.064	0.141	30–40/Female

* Reported responses were for all respondents; ** SD = Strongly disagree, D = Disagree, N = Neutral, A = Agree, SA = Strongly agree.
Reporting forms are not available. I do not know where to report. Reporting is time consuming. All serious ADRs are detected before registration of drug. I do not report ADRs because I want to publish the case by myself. I fear to harm the confidence of my patients. Patients do not tell us about ADRs that they experience after medicine. I have insufficient knowledge in detecting ADRs. I do not know how to report ADRs. There is no pharmacovigilance/ADRs reporting center in Pakistan. I am not convinced that the ADRs are caused by the drug. I am unable to find which drug caused the ADR.

**Table 4:** Factors Encouraging Physician/Pharmacists to Report an ADRs.

	Responses *					*p* Value **			
Statements	SA	A	N	D	SD	Age	Gender	Type of Practice	Direction of Association
	*n* (%)	*n* (%)	*n* (%)	*n* (%)	*n* (%)				
1	40 (22%)	47 (25.8%)	95 (52.2%)	0	0	**0.001**	0.016	**0.001**	30–40/Employee
2	0	87 (47.8%)	47 (25.8%)	48 (26.4%)	0	**0.001**	0.455	**0.001**	30–40/Employee
3	0	95 (52.2%)	87 (47.8%)	0	0	**0.003**	0.174	0.064	<30
4	90 (49.4%)	58 (31.9%)	34 (18.7%)	0	0	**0.001**	0.849	**0.001**	30–40/Employee
5	97 (53.3%)	52 (28.5%)	30 (16.5%)	3 (1.6%)	0	**0.008**	0.189	**0.037**	30–40/Manager
6	47 (25.8%)	47 (25.8%)	88 (48.3%)	0	0	**0.003**	**0.001**	0.181	30–40/Employee

* Reported responses were for all respondents; ** SD = Strongly disagree, D = Disagree, N = Neutral, A = Agree, SA = Strongly agree.
There is an obligation to do so. There are incentives. I see my colleagues doing so. I receive letters for reporting from relevant authorities. There will be a reporting center in our country. If there will be a toll -free number provided by relevant authorities.

## References

[1] World Health Organization | Pharmacovigilance. http://www.who.int/medicines/areas/quality_safety/safety_efficacy/pharmvigi/en/.

[2] McDonnell P.J., Jacobs M.R. (2002). Hospital admissions resulting from preventable adverse drug reactions. Ann. Pharmacother..

[3] Bates D.W., Cullen D.J., Laird N., Petersen L.A., Small S.D., Servi D., Laffel G., Sweitzer B.J., Shea B.F., Hallisey R. (1995). Incidence of adverse drug events and potential adverse drug events: Implications for prevention. JAMA.

[4] Scurti V., Romero M., Tognoni G. (2012). A plea for a more epidemiological and patient-oriented pharmacovigilance. Eur. J. Clin. Pharmacol..

[5] Mahmood K.T., Tahir F., Haq I.U. (2011). Pharmacovigilance: A need for best patient care in Pakistan: A review. J. Pharm. Sci. Res..

[6] Davies E.C., Green C.F., Taylor S., Williamson P.R., Mottram D.R., Pirmohamed M. (2009). Adverse drug reactions in hospital in-patients: A prospective analysis of 3695 patient-episodes. PLoS ONE.

[7] Ramezani Tehrani B., Javid Nikoo N. (2007). Report of the Iranian pharmacovigilance center. Razi J..

[8] Inman W. (1996). Attitudes to adverse drug reaction reporting. Br. J. Clin. Pharmacol..

[9] Desai C.K., Iyer G., Panchal J., Shah S., Dikshit R. (2011). An evaluation of knowledge, attitude, and practice of adverse drug reaction reporting among prescribers at a tertiary care hospital. Perspect. Clin. Res..

[10] Babbie E. (2008). The Basics of Social Research.

[11] Bourque L., Fielder E. (2003). How to Conduct Self-Administered and Mail Surveys.

[12] Herdeiro M.T., Figueiras A., Polonia J., Gestal-Otero J.J. (2006). Influence of pharmacists’ attitudes on adverse drug reaction reporting: A case-control study in Portugal. Drug Saf..

[13] Belton K., Lewis S.C., Payne S., Rawlins M., Wood S. (1995). Attitudinal survey of adverse drug reaction reporting by medical practitioners in the United Kingdom. Br. J. Clin. Pharmacol..

[14] Mes K., Berg L., Grootheest A. (2002). Attitudes of community pharmacists in The Netherlands towards adverse drug reaction reporting. Int. J. Pharm. Pract..

[15] Bateman D., Sanders G., Rawlins M. (1992). Attitudes to adverse drug reaction reporting in the northern region. Br. J. Clin. Pharmacol..

[16] Generali J.A., Danish M.A., Rosenbaum S.E. (1995). Knowledge of and attitudes about adverse drug reaction reporting among Rhode Island pharmacists. Ann. Pharmacother..

[17] Sweis D., Wong I.C. (2000). A survey on factors that could affect adverse drug reaction reporting according to hospital pharmacists in Great Britain. Drug Saf..

[18] Barnett V. (1991). Sample Survey: Principles an Methods.

[19] DeVellis R.F. (2012). Scale Development: Theory and Applications.

[20] Mogey N. (1999). So you want to use a Likert scale. Learning Technology Dissemination Initiative.

[21] Horan P.M., DiStefano C., Motl R.W. (2003). Wording effects in self-esteem scales: Methodological artifact or response style?. Struct. Equ. Model..

[22] Sekaran U. (2003). Research Method for Business: A Skill Approach.

[23] Hinton P.R., McMurray I., Brownlow C. (2014). SPSS Explained.

[24] Dawson B., Trapp R.G., Trapp R.G. (2004). Basic & Clinical Biostatistics.

[25] Kuzon Jr W.M., Urbanchek M.G., McCabe S. (1996). The seven deadly sins of statistical analysis. Ann. Plast. Surg..

[26] Millis S.R. (2003). Statistical practices: The seven deadly sins. Child Neuropsychol..

[27] Jamieson S. (2004). Likert scales: How to (ab) use them. Med. Educ..

[28] Lee K.K., Chan T.Y., Raymond K., Critchley J.A. (1994). Pharmacists’ attitudes toward adverse drug reaction reporting in Hong Kong. Ann. Pharmacother..

[29] Granas A.G., Buajordet M., Stenberg-Nilsen H., Harg P., Horn A.M. (2007). Pharmacists’ attitudes towards the reporting of suspected adverse drug reactions in norway. Pharmacoepidemiol. Drug Saf..

[30] Toklu H.Z., Uysal M.K. (2008). The knowledge and attitude of the Turkish community pharmacists toward pharmacovigilance in the Kadikoy district of Istanbul. Pharm. World Sci..

[31] Williams D., Feely J. (1999). Underreporting of adverse drug reactions: Attitudes of irish doctors. Irish J. Med. Sci..

[32] Green C.F., Mottram D.R., Rowe P.H., Pirmohamed M. (2001). Attitudes and knowledge of hospital pharmacists to adverse drug reaction reporting. Br. J. Clin. Pharmacol..

[33] Green C.F., Mottram D.R., Raval D., Proudlove C., Randall C. (1999). Community pharmacists’ attitudes to adverse drug reaction reporting. Int. J. Pharm. Pract..

[34] Herdeiro M.T., Figueiras A., Polónia J., Gestal-Otero J.J. (2005). Physicians’ attitudes and adverse drug reaction reporting. Drug Saf..

[35] Li Q., Zhang S.-M., Chen H.-T., Fang S.-P., Yu X., Liu D., Shi L.-Y., Zeng F.-D. (2004). Awareness and attitudes of healthcare professionals in Wuhan, China to the reporting of adverse drug reactions. Chin. Med. J..

[36] Hasford J., Goettler M., Munter K.H., Müller-Oerlinghausen B. (2002). Physicians’ knowledge and attitudes regarding the spontaneous reporting system for adverse drug reactions. J. Clin. Epidemiol..

[37] Grootheest V. (1999). Attitudinal survey of voluntary reporting of adverse drug reactions. Br. J. Clin. Pharmacol..

[38] Figueiras A., Herdeiro M.T., Polónia J., Gestal-Otero J.J. (2006). An educational intervention to improve physician reporting of adverse drug reactions: A cluster-randomized controlled trial. JAMA.

[39] Vallano A., Cereza G., Pedros C., Agustí A., Danés I., Aguilera C., Arnau J. (2005). Obstacles and solutions for spontaneous reporting of adverse drug reactions in the hospital. Br. J. Clin. Pharmacol..

[40] Bawazir S.A. (2006). Attitude of community pharmacists in Saudi Arabia towards adverse drug reaction reporting. Saudi Pharm. J..

[41] Irujo M., Beitia G., Bes-Rastrollo M., Figueiras A., Hernández-Díaz S., Lasheras B. (2007). Factors that influence under-reporting of suspected adverse drug reactions among community pharmacists in a Spanish region. Drug Saf..

[42] Obara T., Yamaguchi H., Iida Y., Satoh M., Sakai T., Aoki Y., Murai Y., Matsuura M., Sato M., Ohkubo T. (2016). Knowledge of and perspectives on pharmacovigilance among pharmacists in the Miyagi and Hokkaido regions of Japan. J. Pharmacovigil..

[43] Catic T., Begović B. (2016). The attitudes of pharmacists and physicians in Bosnia and Herzegovina towards adverse drug reaction reporting. J. Health Sci..

[44] Anton C., Cox A.R., Ferner R.E. (2009). Improving follow-up rates in spontaneous adverse drug reaction reporting. Drug Saf..

[45] Al-Worafi Y.M., Kassab Y.W., Alseragi W.M., Almutairi M.S., Ahmed A., Ming L.C., Alkhoshaiban A.S., Hadi M.A. (2017). Pharmacovigilance and adverse drug reaction reporting: A perspective of community pharmacists and pharmacy technicians in Sana’a, Yemen. Ther. Clin. Risk Manag..

[46] Chang F., Xi Y., Zhao J., Zhang X., Lu Y. (2017). A time series analysis of the effects of financial incentives and mandatory clinical applications as interventions to improve spontaneous adverse drug reaction reporting by hospital medical staff in China. J. Eval. Clin. Pract..

[47] Scott E.K., Joan H. (2001). Physician response to surveys: A review of the literature. Am. J. Prev. Med..

